# Funding innovation in neglected diseases

**DOI:** 10.1136/bmj.k1182

**Published:** 2018-03-20

**Authors:** Gavin Yamey, Amie Batson, Peter H Kilmarx, Marcel Yotebieng

**Affiliations:** 1 Center for Policy Impact in Global Health, Duke University, Durham, NC, USA; 2 PATH, Seattle, WA, USA; 3 Fogarty International Center, National Institutes of Health, Bethesda, MD, USA; 4 Division of Epidemiology, Ohio State University, Columbus, OH, USA

## Abstract

Concerted action is required to reverse downward trends in research and development

Neglected diseases are those with insufficient markets to attract investment from the drug industry. They primarily affect populations living in low income countries and include malaria, tuberculosis, and diarrhoeal diseases.

Public and philanthropic funding is required to develop new health technologies to control these diseases. While funding from public-private partnerships helped to bring 37 new treatments for neglected diseases to market between 2000 and 2011, this represented just 4% of all new therapeutic products registered during this period. As neglected diseases cause about 11% of the global burden of disease, there is clearly a “persistent insufficiency” in research and development (R&D).^1^ Except for a one-off injection of funding for Ebola and other African viral haemorrhagic fevers, funding for product development for neglected diseases has shown a downward trend since 2009.^2^

How can this trend be reversed? Strategies to mobilise funding should engage governments from high, low, and middle income countries, philanthropic foundations, and the private sector. We propose five interconnected approaches.

Firstly, the public and philanthropic sectors should continue to expand successful incentive mechanisms and test new ones to attract industry to participate in product development for neglected diseases. These incentives include long term partnerships that provide public investments in translational research and clinical infrastructure. They also include downstream investments, or “pull” funding (the promise of reward for successful drug development), such as guaranteed purchase of products.^3^ ^4^ ^5^ Given the growing need for innovative financing, funders should support experimental approaches, such as the Life Prize, which combines upfront grants with pooling of intellectual property into a patent pool.^6^

Secondly, funders could support a “health investors’ platform” to improve the targeting of limited resources. Poor coordination of investments is a key challenge in financing research for neglected diseases, especially in the private sector. A health investors’ platform would facilitate pooling and sharing of information on R&D needs, candidate products in the pipeline, estimated development costs and financing gaps, likely markets, and expected health and economic benefits. The platform could help to inform public, private, and philanthropic investors—and attract new investors—to fund those candidate products likely to have the largest public health benefits.

The World Health Organization’s new Global Observatory on Health Research and Development, though operating on a shoestring, is a promising first step towards building this kind of platform.^7^ WHO has a critical role in supporting R&D for neglected diseases, through convening experts, prioritising needs, and supporting countries to conduct health technology assessments.^8^ WHO member states need to increase their financial contributions to fully support this work.

A third approach is for international donors to work more closely with experts within low and middle income countries to tackle the documented mismatch between global and national research priorities.^9^ Developing a new framework for shared prioritisation would be a valuable step towards making R&D more “needs driven” and the resulting innovations more scalable. Regional institutions in these countries, such as the Africa Centres for Disease Control and Prevention, could help facilitate this coordination.

Fourthly, many low and middle income countries—such as Brazil, Cuba, India, Indonesia, and South Africa—are already funding research into neglected diseases, and a coordinated global effort is needed to encourage others to follow suit.^10^ In 2016, for example, India was the fourth and Brazil was the ninth largest funder of research into neglected disease globally.^2^ Governments and foundations could support the creation of an international “roadmap” for research—an analysis of the current capacities of all countries, the steps each country should take to increase its capacity, and the costs of such improvement. The roadmap would cover the entire life cycle from laboratory to implementation science and include indicators of progress.

This roadmap could help donors target their support for capacity building in low and middle income countries. It could also be a helpful tool for the new Coalition for African Research and Innovation, an alliance of African science leaders and international funders that aims to build “a highly coordinated, well funded, and African led African innovation enterprise.”^11^ Other initiatives that could benefit from this roadmap are the Coalition for Epidemic Preparedness Innovations, which aims to develop new vaccines for epidemic control^12^; the G20’s collaboration hub for antimicrobial R&D^13^; the European and Developing Countries Clinical Trial Partnership, which has a major focus on building research capacity in Africa^14^; and the US National Institutes of Health’s Fogarty International Center, which builds research capacity in lower income countries.^15^

Finally, donors could help to unlock domestic resources for research in low and middle income countries, such as through a matching fund that pairs global and national resources for shared priorities. The movement towards universal health coverage provides the perfect window of opportunity for these countries’ governments to commit a percentage of domestic health budgets to R&D. Their governments must be in the driving seat in the development of affordable, scalable innovations that will help achieve universal health coverage. Engaging ministries across different sectors and studying the health and economic returns on domestic investment in research could help build political commitment.

Today’s health technologies are insufficient to end the death and suffering caused by neglected diseases. Concerted action on multiple fronts is required to mobilise funding to develop new treatments, to optimise its impact, and to put in place a sustainable pipeline of innovations for these important diseases.

## Notes

Provenance and peer review: Not commissioned, externally peer reviewed.
